# A Bilayer Skin-Inspired Hydrogel with Strong Bonding Interface

**DOI:** 10.3390/nano12071137

**Published:** 2022-03-29

**Authors:** Chubin He, Xiuru Xu, Yang Lin, Yang Cui, Zhengchun Peng

**Affiliations:** Center of Stretchable Electronics and Nanosensors, School of Physics and Optoelectronic Engineering, Shenzhen University, Shenzhen 518060, China; hcbfighting@163.com (C.H.); linyangyy@163.com (Y.L.); cuiqyang@163.com (Y.C.)

**Keywords:** conductive hydrogels, bilayer structure, interface robustness, wearable sensors

## Abstract

Conductive hydrogels are widely used in sports monitoring, healthcare, energy storage, and other fields, due to their excellent physical and chemical properties. However, synthesizing a hydrogel with synergistically good mechanical and electrical properties is still challenging. Current fabrication strategies are mainly focused on the polymerization of hydrogels with a single component, with less emphasis on combining and matching different conductive hydrogels. Inspired by the gradient modulus structures of the human skin, we propose a bilayer structure of conductive hydrogels, composed of a spray-coated poly(3,4-dihydrothieno-1,4-dioxin): poly(styrene sulfonate) (PEDOT:PSS) as the bonding interface, a relatively low modulus hydrogel on the top, and a relatively high modulus hydrogel on the bottom. The spray-coated PEDOT:PSS constructs an interlocking interface between the top and bottom hydrogels. Compared to the single layer counterparts, both the mechanical and electrical properties were significantly improved. The as-prepared hydrogel showed outstanding stretchability (1763.85 ± 161.66%), quite high toughness (9.27 ± 0.49 MJ/m^3^), good tensile strength (0.92 ± 0.08 MPa), and decent elastic modulus (69.16 ± 8.02 kPa). A stretchable strain sensor based on the proposed hydrogel shows good conductivity (1.76 S/m), high sensitivity (a maximum gauge factor of 18.14), and a wide response range (0–1869%). Benefitting from the modulus matching between the two layers of the hydrogels, the interfacial interlocking network, and the patch effect of the PEDOT:PSS, the strain sensor exhibits excellent interface robustness with stable performance (>12,500 cycles). These results indicate that the proposed bilayer conductive hydrogel is a promising material for stretchable electronics, soft robots, and next-generation wearables.

## 1. Introduction

As the new generation of high-performance conductive elastomers, conductive hydrogel is widely used in sports monitoring, healthcare, electronic skins, energy storage devices, and other fields [1,2,3,4,5,6,7]. However, due to the single and loose gel network of traditional synthetic hydrogels and the lack of efficient energy dissipation between the molecular chains, they often exhibit brittle and weak mechanical properties under external forces [8,9], which greatly limits their application development. Researchers have taken great efforts in improving the mechanical performances of conductive hydrogels. For example, Zhang and co-workers developed a conductive hydrogel mechanoreceptor with tensile strain of 1731%, modulus about 22.9 kPa, and toughness of 1.4576 MJ/m^3^, by constructing the adenosine monophosphate cross-linked quaternized chitosan network and sodium chloride-containing polyacrylamide frameworks [10]. Tong and co-workers obtained a physically linked three-dimensional hierarchical functionalized-boron nitride/clay/poly(N-isopropylacrylamide) ternary network hydrogel by utilizing the functionalized boron nitride nanosheets to introduce readily reformable non-covalent bonds as crosslinkers and sacrificial bonds, which showed a large ultimate strain of 980% at a tensile strength of 40 kPa [11]. Nevertheless, achieving a conductive hydrogel with skin-like softness, good ductility, excellent tensile strength, and high toughness is still challenging. The human skin has several layers—the epidermis layer, the dermis layer, and the subcutaneous tissue layer. The dermis layer, particularly, is observed to have gradient modulus structures with both low modulus and high modulus layers, which offers the skin elasticity, impact resistance and sensitivity, among other functions. Therefore, inspired by different modulus structures of the human skin, two different types of hydrogels with different modulus and other functions can be combined together [12,13,14]. To achieve this goal, there are two crucial issues that need to be considered: (1) The matching of both modulus and fabricating process between the two candidate layers. (2) The stability issues in long-term use. The building blocks of the interface between the relatively lower and higher modulus layers are crucial for working performances, such as reliability and stability.

Here, inspired by the human skin, we successfully prepared a bilayer conductive hydrogel using the layer-by-layer polymerization process. According to the advantages of gradient modulus structures, we combined a relatively higher modulus (hard) hydrogel and a relatively lower modulus (soft) hydrogel. In this work, polyacrylamide (PAAm), poly(N-methylol) acrylamide (PNMA), and polyvinyl pyrrolidone (PVP) were studied as the polymer framework. We then used potassium chloride (KCl) as an ionic conductive agent, carboxylated cellulose nano-whiskers (C-CNWs) as a physical cross-linking nano-enhancer, ammonium persulfate (APS) as the thermal initiator, and acrylamide grafted xanthan gum (XG) as the chemical cross-linker. Then, two monolayer hydrogels were prepared, separately—the PAAm/PANC-CNWs (PANC) hydrogel with high modulus and the PAAm/PVP-KCl (PAVK) hydrogel with low modulus. Poly(N-methylol) acrylamide (PNMA) and polyvinyl pyrrolidone (PVP) can be used to adjust the polymer matrix. Subsequently, we used PEDOT:PSS as the interfacial layer, which is a high-performance and highly conductive proton-doped conductive polymer, to composite the two layers of hydrogels to prepare PSS–PANC conductive hydrogels with a bilayer structure. The pre-polymerization and spray-coating techniques were used to fabricate the interlocking building blocks between the two layers. Two matching groups of bilayer hydrogels were compared. Moreover, the mechanical interface robustness, fatigue resistance, and electrical working stability were demonstrated to have been significantly improved by introducing the interface of PEDOT:PSS.

## 2. Materials and Methods

### 2.1. Preparation of Monolayer and Bilayer Conductive Hydrogels

All the reagents were purchased from Aladdin Chem Shanghai (China) and used as received without any further purification. First, we prepared three types of monolayer hydrogels.

➀Polyacrylamide (PAAm)/poly(N-methylol) acrylamide (PNMA)/carboxylated cellulose nano-whiskers (C-CNWs) hydrogel (named as PANC hydrogel with high modulus): We added 5.00 g deionized water (DI water), 1.20 g acrylamide monomer (AAm), 0.05 g N-hydroxymethyl acrylamide (NMA), and 0.10 g carboxylated cellulose nano-whiskers (C-CNWs) into a glass beaker. Subsequently, continuous stirring was carried out at 70 °C for 4 h to completely dissolve all the substances. After cooling down to room temperature (25 °C), we weighed out 0.03 g of ammonium persulfate (APS), centrifuged it to remove bubbles, poured an appropriate amount of the precursor solution into the template, and placed it into an oven for 45 min at 80 °C.

➁Polyacrylamide (PAAm)/poly(N-methylol) acrylamide (PNMA)/carboxymethyl chitosan (CMCS)–calcium chloride (CaCl_2_) hydrogel (named as PANS hydrogel with high modulus): Similarly, we weighed 5.00 g DI water, 1.00 g AAm monomer, and 0.50 g carboxymethyl chitosan (CMCS) into a glass beaker. The temperature was increased to 80 °C and stirred for 3 h. Then, 0.10 g calcium chloride (CaCl_2_) were added to the precursor solution. After CaCl_2_ was dissolved, the temperature was lowered down to room temperature. Subsequently, 0.10 g NMA and 0.03 g APS were weighed in sequence and dissolved evenly. After centrifuging to remove bubbles, the uniform solution was poured into the template and placed in an oven for 45 min at 80 °C.

➂Polyacrylamide (PAAm)/polyvinyl pyrrolidone (PVP)/potassium chloride (KCl) hydrogel (named as PAVK hydrogel with low modulus): Separately, we weighed 1.20 g of AAm monomer, 0.10 g of PVP, 0.03 g of potassium chloride (KCl), 0.001 g of acryl-grafted xanthan gum, and 5.00 g of DI water; the mixture was completely dissolved for 2 h. We then weighed out 0.03 g APS and centrifuged it to remove bubbles. Next, we poured the precursor mixer into the template and placed it into an oven for 45 min at 80 °C.

Secondly, we prepared three types of bilayer hydrogels. Using the obtained precursor mixers of PANS and PANC hydrogels, we poured the PANS and the PANC precursor mixer into the template and placed it into an 80 °C oven for a 7 min pre-polymerization treatment, respectively. The pre-polymerized PANS and PANC hydrogel layers were then obtained. Next, we transferred poly(3,4-dihydrothieno-1,4-dioxin): poly(styrene sulfonate) (PEDOT:PSS) aqueous dispersion (Clevios P, Heraeus, Germany) to a spray gun and sprayed the PEDOT:PSS aqueous dispersion onto the PANS and PANC pre-polymerized hydrogel layers to form the PEDOT:PSS interfacial layers, respectively. Finally, we applied the as-prepared PAVK precursor mixer onto the surface of the PEDOT:PSS treated PANS and PANC hydrogels. After 80 °C in the oven for 42 min, they were taken out and cooled down to room temperature. Then, the PAVK-PEDOT:PSS-PANS and PAVK-PEDOT:PSS-PANC bilayer hydrogels were well prepared. We also used a similar pre-polymerization process to fabricate the PAVK/PANS and PAVK/PANC bilayer hydrogels samples without the spray-coating process of the PEDOT:PSS interface layer.

### 2.2. Fabrication of Strain Sensors from Conductive Hydrogels

The as-prepared bilayer conductive hydrogel was cut into a rectangular shape with a size of 20 mm × 5 mm × 1 mm. Subsequently, we used copper wires to connect the tailored bilayer conductive hydrogel and fixed it to a 3M “very high bond” (VHB) tape for 24 h to manufacture the hydrogel strain sensors.

### 2.3. Characterization and Tests

The light transmittance of each hydrogel sample was tested using an ultraviolet/visible/near-infrared spectrophotometer (UV-vis, PerkinElmer Lambda 950, Waltham, MA, USA). The testing range was 300–700 nm, and the scan rate was 500 nm/min. The infrared spectrum was measured by a Fourier transform infrared spectrometer (FT-IR) with diffuse reflection (model of Spectrum One Version, PerkinElmer, Inc., Waltham, USA). The scanning frequency range was 4000–400 cm^−1^. The electrical conductivity of the gel samples was tested by a digital multimeter (34465A, Keysight, Guangzhou, China). The mechanical properties were performed by a universal stretching machine (E1000, Instron, Boston, MA, USA), where the tensile test rate was 100 mm/min. The T-peel test was carried out by E1000 Instron with a test rate of 5 mm/min.

## 3. Results and Discussion

**Figure 1:**
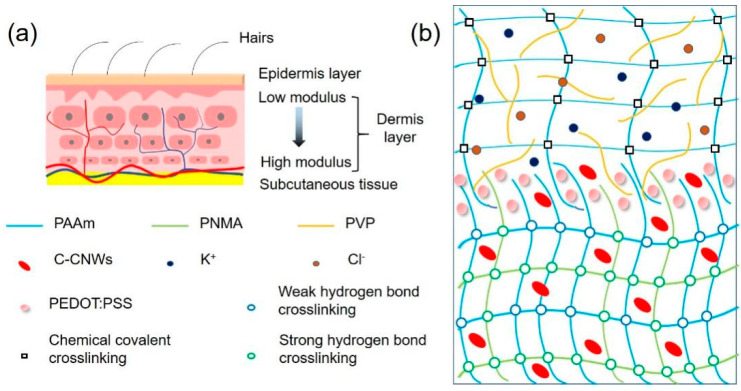
Schematic structure of (**a**) human skin and (**b**) the interfacial PEDOT:PSS enhanced bilayer conductive hydrogel.

The skin is the largest organ in the human body. It serves as the strongest protective layer for human tissues, and can transmit substances (such as nutrients, heat, and sweat) to the human body. Moreover, the skin is a multifunctional sensor that can sense the external environment (such as temperature and stress). As shown in Figure 1a, the skin is a layered structure with gradual modulus [15,16]. The epidermal layer is the outermost layer, which has good ductility and sufficient toughness to protect the skin from cracking when deformed. The next layer is the dermis layer, composed of rich elastic and collagen fibers. The layer-by-layer stacking of nano-biofibers gives the dermis a gradual change in modulus [17]. This change in modulus gives the skin good elasticity and enables it to support load. Inspired by the multi-layer modulus gradual structure of human skin, we used a relatively high modulus (hard) hydrogel as the support layer, and a relatively low modulus (soft) hydrogel as the conductive response layer and combined both to form a bilayer conductive hydrogel with matching modulus (Figure 1b). Next, we fabricated a conductive PEDOT:PSS interface layer to enhance the interfacial strength and stability of the bilayer hydrogels. By constructing the bilayer hydrogels with both low and high modulus hydrogel layers and improved bonding interfaces, the resultant bilayer hydrogels are expected to improve the mechanical shortcomings of soft gels that were too soft and fragile with low toughness, and hard gels that were tough but with quite low elasticity. Moreover, it will improve the stability performance for long-term use, as well as strengthen the response linear range and cycle stability.

**Figure 2:**
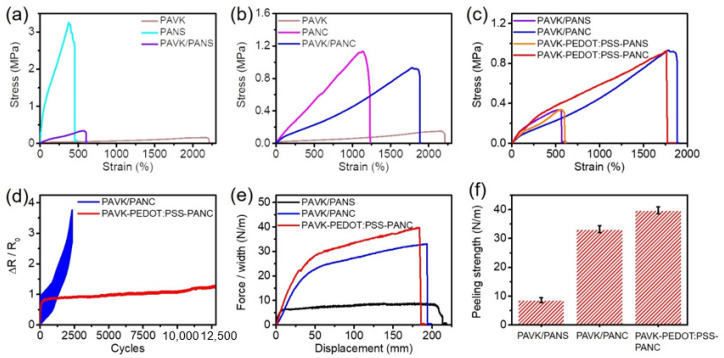
(**a**–**c**) Stress–strain curves of high modulus PANS, high modulus PANC and low modulus PAVK monolayer hydrogels, and PAVK/PANS, PAVK/PANC, and PAVK-PEDOT:PSS-PANC bilayer hydrogels. (**d**) The cyclic tensile response of the PAVK/PANC and PAVK-PEDOT:PSS-PANC bilayer hydrogels. (**e**) The T-peeling force plots and (**f**) peeling strength of PAVK/PANS, PAVK/PANC, and PAVK-PEDOT:PSS-PANC bilayer hydrogels.

First, we synthesized a double-network high modulus PANS hydrogel as a candidate for the support layer by constructing carboxymethyl chitosan (CMCS) and the calcium chloride (CaCl_2_) crosslinking network and the poly(acrylamide-co-N-methylol acrylamide) (P(AAm-co-NMA)) physical crosslinking network. We then formed an ion-conducting low modulus PAVK hydrogel on the top side of the pre-polymerized support hydrogel by using acrylamide grafted xanthan gum as the chemical cross-linker to cross-link PAAm and the polyvinyl pyrrolidone (PVP) network and introduce potassium chloride (KCl) to increase ionic conductivity. However, the as-prepared PANS/PAVK bilayer conductive hydrogel exhibited weak mechanical properties (Figure 2a), such as low stretchability (607.21 ± 7.81%), and toughness (1.15 ± 0.035 MJ/m^3^), which may be due to the mismatch of the elastic modulus of the two layers of hydrogels. As shown in Table 1, the individual elastic modulus ratio between high modulus PANS (1590 ± 6.86 kPa) and low modulus PAVK (31.13 ± 6.30 kPa) was as high as 62 times. To solve this problem, we used carboxylated cellulose nano-whiskers (C-CNWs) to modify the P(AAm-co-NMA) physical crosslinking hydrogel framework; the improved hydrogel was named the high modulus PANC hydrogel. The cellulose nano-whiskers C-CNWs can form a tension network with better load-bearing performance due to intramolecular hydrogen bond interactions and can also form multiple intermolecular hydrogen bond interactions between polymer chains (Figure 2b and Table 1). Compared to PAVK/PANS bilayer conductive hydrogels, PAVK/PANC bilayer conductive hydrogels showed better mechanical properties and modulus adaptability (Figure 2c). The individual elastic modulus ratio between high modulus PANC (137.77 ± 9.29 kPa) and low modulus PAVK (31.13 ± 6.30 kPa) was reduced to about 4.4. The as-prepared bilayer PAVK/PANC showed excellent mechanical performance, fracture strain of 1882.18 ± 193.16%, tensile stress of 0.94 ± 0.022 MPa, elastic modulus of 83.13 ± 6.44 kPa, and toughness of 8.61 ± 0.26 MJ/m^3^. This indicated that modification of the high modulus PANC hydrogel with cellulose nano-whiskers C-CNWs significantly improved the elastic modulus matching between the high modulus PANC hydrogel and the low modulus PAVK.

However, without any interface treatments, when we tested the cyclic tensile response of the PAVK/PANC bilayer conductive hydrogel, we found poor stability with less than 2500 cycles tolerance (Figure 2d). This poor stability may greatly limit the application of the bilayer hydrogels for practical usage. It is essential to improve the interfacial stability of the bilayer hydrogels. Therefore, we further constructed an interface layer between the bilayers of the hydrogel to enhance the interfacial strength. Briefly, we fabricated a pre-polymerized PANC monolayer hydrogel as the supporting layer, and then spray-coated a PEDOT:PSS interface layer on the PANC hydrogel. Next, the PAVK hydrogel precursor was applied on the top, and after heating and curing, we obtained the PAVK-PEDOT:PSS-PANC bilayer hydrogel. The cyclic tensile response test results showed that the PAVK-PEDOT:PSS-PANC bilayer hydrogel can stably respond to more than 12,000 tensile loading–unloading cycles (Figure 2d), indicating that the treating of the PEDOT:PSS interface layer further improved the interfacial strength, adhesion, and stability of the PAVK/PANC bilayer conductive hydrogel interface.

In order to verify the interfacial stability of the bilayer conductive hydrogels, we measured the interfacial strength of each bilayer hydrogel by a T-peel test. As shown in Figure 2e,f, the peeling strength of PAVK/PANC, without PEDOT hydrogels, was up to 34 N/m, which was about 3.5 times higher than the PAVK/PANS hydrogel (~10 N/m). This indicated that the addition of cellulose nano-whiskers C-CNWs also significantly improved the interfacial strength of the PAVK/PANC bilayer conductive hydrogel. This may be due to the elastic modulus matching between low modulus PAVK and high modulus PANC, as well as the formation of effective interactions at the interface area, which is a tighter interfacial interlocking network structure by intramolecular and intermolecular hydrogen bonding interactions formed between nano-whiskers C-CNWs (rich of -COOH and -OH groups on the surface) and the polymer chains in the hydrogel. Furthermore, after applying the PEDOT:PSS interface layer, the interfacial strength of the PAVK/PANC bilayer hydrogel was further enhanced, reaching ~40 N/m (Figure 2f), which indicated that the treatment of the PEDOT:PSS interface layer can effectively enhance the interfacial toughness and interfacial strength of the PAVK/PANC bilayer conductive hydrogel, and the PEDOT:PSS treated bilayer hydrogel showed excellent mechanical properties, with fracture strain of 1763.85 ± 161.66%, tensile strength of 0.92 ± 0.076 MPa, elastic modulus of 69.16 ± 8.02 kPa, and toughness of 9.27 ± 0.49 MJ/m^3^ (Table 1). Additionally, we also fabricated the PAVK-PEDOT:PSS-PANS bilayer hydrogels. Compared to the performance of the PAVK/PANS bilayer hydrogel (607.21 ± 7.81% of fracture strain, 1.15 ± 0.035 MJ/m^3^ of toughness), the PAVK-PEDOT:PSS-PANS bilayer hydrogel also showed a similar quite low fracture strain of 568.36 ± 10.71% and a slightly increased toughness of 1.24 ± 0.17 MJ/m^3^. For the PAVK-PEDOT:PSS-PANS bilayer hydrogel, after treating with the PEDOT:PSS interfacial layer, the toughness was slightly improved but still with poor interface stability. For the absence of cellulose nano-whiskers, only treatment with PEDOT:PSS is not enough to construct a strong enough interlocking network interface structure, and there is a significant mismatch of the elastic modulus between the low modulus PAVK and high modulus PANS hydrogels.

**Figure 3:**
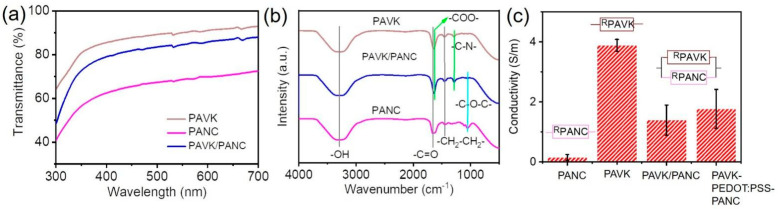
(**a**) UV−vis spectra, (**b**) FT-IR spectroscopy, and (**c**) conductivity of individual monolayer and bilayer conductive hydrogels.

We characterized the physical properties of the PAVK/PANC bilayer conductive hydrogels. All the bilayer conductive hydrogels showed a quite high optical transparency (Figure 3a). From the ultraviolet-visible wavelength of 300 to 700 nm, the light transmittance of the PAVK/PANC bilayer conductive hydrogel reached over 88%. Further, we performed FT–IR characterization on different hydrogel samples. The characteristic peaks at 3300, 1653, and 1454 cm^−1^ represented -OH, -C=O, and -CH_2_-CH_2_- stretching vibration, respectively (Figure 3b) [18,19]. For the PANC monolayer hydrogel, the stretching vibration peak of the C-O-C groups on C-CNWs also appeared at 1051 cm^−1^ [20]. As for the PAVK monolayer hydrogel, the characteristic peaks of the stretching vibration of the carboxyl C=O bonds (-COO-) from xanthan gum and the C-N groups from PVP polymer chains appeared at 1633 and 1286 cm^−1^, respectively [21]. These characteristic peaks were all found in the bilayer conductive hydrogel without observable shifts, which showed that C-CNWs, PVP, and other components were successfully introduced into the gel network of the PAVK/PANC bilayer conductive hydrogel. Further, we tested the conductivity of each monolayer and bilayer conductive hydrogels (Figure 3c). The PAVK monolayer hydrogel showed a larger ionic conductivity with a value of 3.88 S/m, owing to the introduction of KCl. In contrast, the conductivity of the PANC monolayer hydrogel was only 0.15 S/m, which can be further improved by adding conductive KCl and other salts. The conductivity of the PAVK/PANC and PAVK-PEDOT:PSS-PANC bilayer were 1.39 and 1.76 S/m, respectively. The conductivity value of the PAVK-PEDOT:PSS-PANC was equivalent to the result of the low modulus PAVK and high modulus PANC hydrogels parallel connected in the circuit. The conductivity of the PAVK-PEDOT:PSS-PANC bilayer hydrogel was higher than that without PEDOT:PSS, indicating that the introduction of the PEDOT:PSS interface layer improved the ion transport between the top and bottom hydrogels.

**Figure 4:**
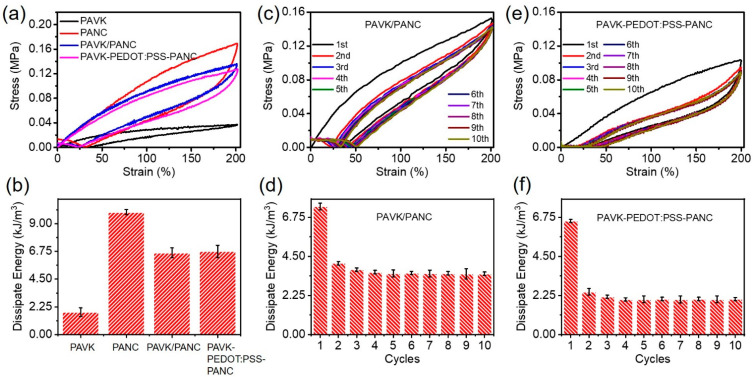
(**a**) The cyclic loading–unloading tests and (**b**) calculated dissipation energy of low modulus PAVK, and high modulus PANC monolayer hydrogels and PAVK/PANC, and PAVK-PEDOT:PSS-PANC bilayer hydrogels, respectively. (**c**) The 10 cycles tensile test and the corresponding dissipate energy for each cycles of (**c**,**d**) PAVK/PANC and (**e**,**f**) PAVK-PEDOT:PSS-PANC bilayer hydrogels.

Next, we carried out cyclic loading–unloading tests on different hydrogels to study the interfacial toughness and energy dissipation of the conductive hydrogels (after a 100 cycle 0–200% strain loading–unloading training to stabilize the performance of the hydrogels), and calculated the corresponding dissipation energy. As shown in Figure 4a, all hydrogel samples showed obvious hysteresis circles in the strain range of 0–200%. Among them, the PANC monolayer hydrogel showed the largest hysteresis circle, and its dissipation energy was also the largest, reaching 9.94 kJ/m^3^ (Figure 4b). This was because its frame polymer PNMA can form strong hydrogen bonds with polymer PAAm and cellulose nano-whiskers C-CNWs, and the -COOH and -OH groups on the surface of C-CNWs can form multiple dynamic hydrogen bonds with the matrix (Figure 4a,b). This was because the acryl-grafted xanthan gum (acryl-grafted XG) acted as a chemical cross-linking agent to form a good paddy chemical cross-linking network with PAAm. Furthermore, the dissipation energy of the bilayer conductive hydrogel without and with the PEDOT:PSS interface layer sits in the middle—between the PANC monolayer hydrogel and the PAVK monolayer hydrogel (Figure 4b). The reasons for this can be concluded as follows: first, the bilayer conductive hydrogel inherits and retains the original dissipation mechanism of each single-layer hydrogels. For example, acryl-grafted xanthan gum can form a chemical cross-linking network with PAAm; thus, the bilayer hydrogel shows good elasticity and recovery. Meanwhile, a large number of multiple hydrogen bonds was formed between PNMA, C-CNWs, PVP, and the matrix, thereby improving the tensile strength and fracture toughness of the bilayer conductive hydrogel. Secondly, a strong and stable interface interlocking network structure was formed in the bilayer hydrogel (Figure 1) [13,14]. Due to the porous network in the hydrogel, a part of the PAVK hydrogel precursor can be penetrated into the PANC hydrogel, which can be polymerized with the PANC substrate to form chemical cross-linking sites. Moreover, due to the presence of polymers such as C-CNWs and PVP as the precursor, multiple hydrogen bonds can be formed between them; therefore, the interlocking network structure with chemical crosslinks and physical interactions can be formed at the interface. The interlocking layer connects the double-layer network firmly and effectively, avoiding the sliding and displacement between the two layers under severe deformation [12,14]. The introduction of the PEDOT:PSS interface layer was more like a “patch”, making the interfacial interlocking network structure of the bilayer conductive hydrogel stronger and more stable [22,23,24].

To characterize the fatigue resistance of the bilayer conductive hydrogels, the bilayer conductive hydrogels with and without the PEDOT:PSS interface layer were subjected to a cyclic tensile test without resting time. It was revealed that after the first stretching cycle, the internal network of all the hydrogels was significantly weakened. The hysteresis curve and dissipation energy of the first cycle were significantly higher than the other cycles, and the hysteresis curve (Figure 4c,e) and dissipation energy tended to be stable (Figure 4d,f). Compared to the bilayer conductive hydrogel without the PEDOT:PSS interface layer, the PAVK-PEDOT:PSS-PANC bilayer hydrogel showed lower dissipation energy and better fatigue resistance, which further indicated that the introduction of the PEDOT:PSS interface layer can improve the interface stability and network structure of the bilayer hydrogel. Therefore, the main reasons for improving the interfacial strength and stability of the bilayer hydrogels were the matching of the upper and lower hydrogel moduli, the tighter interfacial interlocking network structure, and the patch effect of the PEDOT:PSS interface layer.

**Figure 5:**
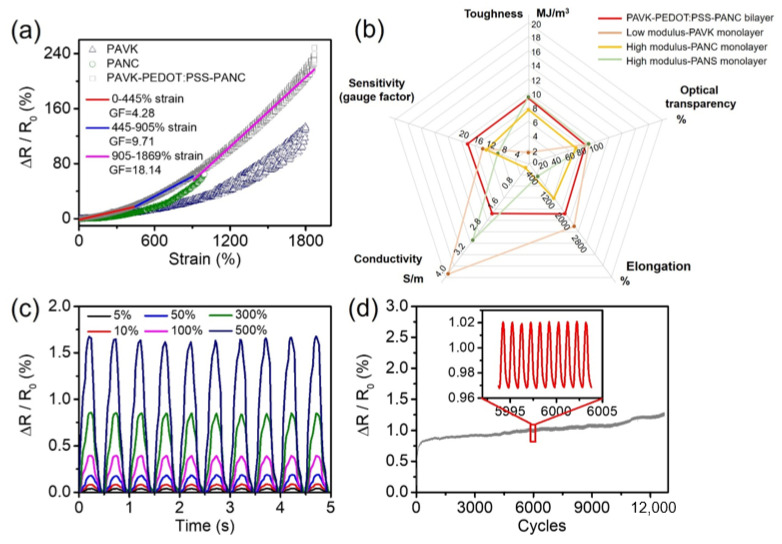
(**a**) The strain response with fitting plots, (**b**) the comparison of comprehensive properties among the low modulus PAVK monolayer, the high modulus PANC monolayer, the high modulus PANS monolayer, and the PAVK-PEDOT:PSS-PANC bilayer hydrogels. (**c**) Cyclic test under different strains and (**d**) cyclic stability at the strain of 200% for the PAVK-PEDOT:PSS-PANC bilayer hydrogel.

Subsequently, we conducted strain-sensing tests on the low modulus PAVK monolayer, high modulus PANC monolayer, and the PAVK-PEDOT:PSS-PANC bilayer hydrogels. Figure 5a shows the relationship between the applied strain and the relative resistance change of the samples during the stretching process; the curve was linearly fitted. From the results, the PAVK-PEDOT:PSS-PANC bilayer hydrogel can respond to a huge strain to 1500% and has an excellent wide linear response range (0–445%). The gauge factor (GF) was 4.28 for the strain from 0 to 445%. The GF value increased to 9.71 for the strain from 445 to 905% and the GF value reached the maximum of 18.14 when the strain was up to 1869%, which was much higher than that of the low modulus PAVK (GFmax = 13.64) and the high modulus PANC (GFmax = 12.59) monolayer hydrogels. The bilayer hydrogel also exhibited a larger measurement range than the monolayer counterparts. We believe the mechanism behind these high performances is as follows: The low modulus PAVK hydrogel, the PEDOT:PSS interfacial layer, and the high modulus PANC hydrogel act as a parallel connection in the equivalent circuit. The conductive networks at the interfacial region allow for relatively easy slippage under stretching. As the stretching continues, the interfacial molecular networks are further pulled out, inducing the interlocking of PEDOT:PSS with both the high modulus PANC hydrogel layer and the low modulus PANS hydrogel layer. With the formation of robust interlocking structures, the PEDOT:PSS maintains strong electronic activity that facilitates charge transfer across the bi-layer. As such, the hydrogel can maintain an effective resistive response in a large strain range. The properties summary of the low modulus PAVK monolayer, high modulus PANC monolayer, high modulus PANS monolayer, and the PAVK-PEDOT:PSS-PANC bilayer hydrogels is shown in Figure 5b. In order to verify the stability of the electrical signal of the hydrogel under multiple mechanical strains, the relative resistance changes of the hydrogel in the repeated stretching process of small strain (5–10%) and large strain (50–500%) were tested. The relative resistance changes of the PANC-PEDOT:PSS-PAVK bilayer hydrogel showed a stable response peak shape under both small and large strains (Figure 5c), indicating that the hydrogel had good repeatability and high sensitivity. Over 12,500 load–unload tensile cycles from 0 to 200% strain were performed in Figure 5d. The experimental results showed that the PAVK-PEDOT:PSS-PANC bilayer hydrogel had excellent cycle stability and durability.

## 4. Conclusions

In summary, we successfully prepared a PANC-PEDOT:PSS-PAVK hydrogel with a bilayer structure, which composed of a spray-coated PEDOT:PSS as the bonding interface. Benefiting from the interlocking networks formed by intramolecular and intermolecular hydrogen bond interactions between the cellulose nano-whiskers C-CNWs and polymer matrix, along with the patch effect of the PEDOT:PSS, the as-prepared hydrogel showed excellent interface robustness and good fatigue resistance. Comparing with the hydrogels without the PEDOT:PSS interface, as well as the single-layered counterparts, both the mechanical and electrical properties of our PANC-PEDOT:PSS-PAVK hydrogel were significantly improved. For instance, our hydrogel exhibited an elongation of 1763.85 ± 161.66%, tensile stress of 0.92 ± 0.08 MPa, toughness of 9.27 ± 0.49 MJ/M^3^, elastic modulus of 69.16 ± 8.02 kPa, conductivity of 1.76 S/m, and over 12,500 cycles of stretching stability. This study not only addresses an interface building block for the development of high-performance conducting hydrogels, but also offers a promising avenue for next-generation wearable electronics and soft robots.

## Figures and Tables

**Table 1:** Summary of the mechanical performances of PAVK, PANS PANC monolayer hydrogels, and PAVK/PANS, PAVK/PANC, and PAVK-PEDOT:PSS-PANC bilayer hydrogels.

Hydrogel Samples	Fracture Strain (%)	Tensile Strength (MPa)	Elastic Modulus (kPa)	Toughness (MJ/m^3^)
Low modulus PAVK monolayer	2208.75 ± 364.52	0.17 ± 0.12	31.13 ± 6.30	1.56 ± 0.32
High modulus PANS monolayer	449.77 ± 168.45	3.28 ± 0.05	1590 ± 6.86	9.47 ± 0.37
High modulus PANC monolayer	1228.79 ± 115.11	1.14 ± 0.05	137.77 ± 9.29	7.63 ± 0.77
PAVK/PANS bilayer	607.21 ± 7.81	0.36 ± 0.005	37.81 ± 4.28	1.15 ± 0.035
PAVK/PANC bilayer	1882.18 ± 193.16	0.94 ± 0.022	83.13 ± 6.44	8.61 ± 0.26
PAVK-PEDOT:PSS-PANS bilayer	568.36 ± 10.71	0.32 ± 0.04	171.38 ± 5.68	1.24 ± 0.17
PAVK-PEDOT:PSS-PANC bilayer	1763.85 ± 161.66	0.92 ± 0.08	69.16 ± 8.02	9.27 ± 0.49

## Data Availability

The data presented in this study are available on request from the corresponding author.
